# Evaluation of a vaccination seminar in regard to medical students' attitudes and their theoretical and practical vaccination-specific competencies

**DOI:** 10.3205/zma001331

**Published:** 2020-06-15

**Authors:** Vera Rill, Björn Steffen, Sabine Wicker

**Affiliations:** 1 Universitätsklinikum Frankfurt, Betriebsärztlicher Dienst, Frankfurt a. M., Germany; 2 Universitätsklinikum Frankfurt, Zentrum der Inneren Medizin, Hämatologie und Onkologie, Frankfurt a. M., Germany

**Keywords:** vaccination certificate, immunization seminar, medical students, attitude, knowledge, practical competence, medical education

## Abstract

**Aim: **Despite having a generally positive attitude toward vaccinations, medical students show gaps in their own immunization histories and knowledge about vaccinations. Future practicing physicians will be confronted with the need to evaluate protective immunity and make vaccination recommendations. This study aims to investigate the extent to which a seminar on the topic of vaccination can improve students’ attitudes, knowledge and practical skills in interpreting vaccination certificates.

**Project description:** Two different one-hour seminars were developed and integrated into the required clinical curriculum. A third of the students attended a theory-based seminar; the other two-thirds completed a predominantly practice-based seminar. The theoretical seminar consisted of a lecture on the principles and theoretical aspects of immunization. In the practical seminar, the curricular content was case-based and taught using fictive examples of vaccination certificates. Before the seminar was held, a voluntary and anonymous survey of the students was conducted regarding their attitudes toward and knowledge of immunization. At the conclusion of the seminar, the students’ ability to understand vaccination certificates was tested. After completing the seminar, all of the participants received a link to participate in a voluntary online survey to evaluate the seminar.

**Results: **Of the 149 seminar attendees in the 2017/18 winter semester, 148 participated in the study.

*Attitude:* Students have a positive attitude toward vaccinations. Regardless of the type of seminar attended, the agreement with statements on vaccination could be significantly increased primarily among students who already at the start of the seminar expressed a high degree of agreement. Students vaccinated against influenza showed significantly stronger agreement than unvaccinated students.

*Knowledge:* Regardless of teaching format, students’ knowledge about vaccination topics could be increased. For those vaccinated against influenza, the mean value for agreement with the statement, *“The vaccination of healthcare workers prevents nosocomial transmission of diseases,”* saw an increase on a five-point Likert scale from 3.97 to 4.4 (p<0.001; R=0.67). For the unvaccinated students, the mean value rose from 4.04 to 4.19 (p=0.06; R=0.29).

*Practical skills:* The students who attended the theory-based seminar tended to score higher on interpreting vaccination certificates than those who attended the practical seminar; however, this difference was not statistically significant.

*Seminar evaluation:* The online evaluation was completed by 18% of the participants. The theoretical seminar received the grade of 2.9 based on the conventional German academic grading scale; the practical seminar received 1.9. This difference is statistically significant (p=0.02).

**Conclusion: **Precisely for skeptical students it was only possible to minimally change existing views with a seminar that offers very brief instruction. Attendees of the theoretical seminar tended to score somewhat higher on interpreting vaccination certificates than those who took the practical seminar. The practical seminar was rated significantly better on the course evaluation than the theoretical one. The advantage that the students attending the theoretical seminar had can be explained best by the structured review of the current vaccination recommendations as part of the seminar, which should, as a consequence, be integrated into the practical seminar.

## Zusammenfassung

**Zielsetzung: **Trotz der generell positiven Einstellung von Medizinstudierenden gegenüber Impfungen sind ihre Impfquoten und ihr Wissen zu Impfungen lückenhaft. In der ärztlichen Praxis werden die zukünftigen ÄrztInnen mit der Beurteilung von Impfschutz und Impfempfehlung konfrontiert sein. In dieser Studie sollte untersucht werden, inwieweit ein Seminar zum Thema Impfen Einstellungen, Wissen und praktische Fertigkeiten der Studierenden im Umgang mit dem Impfpass verbessern kann.

**Projektbeschreibung:** Zwei verschiedene einstündige Impfseminare wurden entwickelt und in das klinische Pflichtcurriculum integriert. Ein Drittel der Studierenden besuchte ein theoretisches Seminar, die verbleibenden zwei Drittel absolvierten ein überwiegend praktisch aufgebautes Seminar. Das theoretische Seminar bestand aus einem Vortrag zu Grundlagen und theoretischen Aspekten des Impfens. Im praktischen Seminar wurden die Lehrinhalte fallbezogen anhand fiktiver Beispiel-Impfpässe vermittelt. Vor Seminarbeginn erfolgte eine freiwillige und anonyme Befragung der Studierenden zu Einstellungen und Wissen zum Thema Impfungen, am Kursende wurden die Fähigkeiten im Umgang mit dem Impfpass überprüft. Nach Abschluss des Kurses wurde an alle Teilnehmer ein Link zu einer freiwilligen Online-Evaluation des Kurses versendet, in der die Studierenden den Kurs bewerten sollten.

**Ergebnisse: **Von den 149 SeminarteilnehmerInnen des Wintersemesters 2017/2018 nahmen 148 an der Studie teil.

*Einstellung: *Studierende sind Impfungen gegenüber positiv eingestellt. Unabhängig vom besuchten Kursformat konnte die Zustimmung zu Impfthemen vor allem bei Studierenden, die bereits zu Seminarbeginn einen hohen Zustimmungsgrad aufwiesen, signifikant gesteigert werden. Gegen Influenza geimpfte Studierende wiesen eine signifikant höhere Zustimmung auf als nicht geimpfte.

*Wissen: *Unabhängig vom Lehrformat konnte das Wissen der Studierenden zu Impfthemen gesteigert werden. In der Gruppe der Influenza-geimpften Studierenden stieg der Mittelwert der Zustimmung zur Aussage *„Die Impfung von Gesundheitspersonal verhindert die nosokomiale Übertragung von Krankheiten“* auf einer fünfstufigen Skala vom Likert-Typ von 3,97 auf 4,4 (p<0,001; R=0,67). Bei den nicht geimpften Studierenden stieg der Mittelwert von 4,04 auf 4,19 (p=0,06; R=0,29).

*Praktische Fähigkeiten: *Die TeilnehmerInnen des theoretischen Seminars schnitten im Umgang mit dem Impfpass tendenziell besser ab als die TeilnehmerInnen des praktischen Seminars, der Unterschied war jedoch nicht statistisch signifikant.

*Kursevaluation: *Die Online-Evaluation wurde von 18% der TeilnehmerInnen abgeschlossen. Das theoretische Seminar wurde im Durchschnitt mit der Schulnote 2,9 bewertet; das praktische Seminar mit der Note 1,9. Dieser Unterschied ist statistisch signifikant (p=0,02).

**Schlussfolgerungen: **Gerade bei skeptisch eingestellten Studierenden können bestehende Ansichten durch ein Seminar mit recht kurzer Unterrichtsdauer nur geringfügig verändert werden. TeilnehmerInnen des theoretischen Seminars schnitten im Umgang mit dem Impfpass tendenziell etwas besser ab als die TeilnehmerInnen des praktischen Seminars. Das praktische Seminar wurde signifikant besser bewertet als das theoretische. Der Vorsprung der TeilnehmerInnen des theoretischen Seminars im Bereich der praktischen Fertigkeiten erklärt sich am ehesten durch die strukturierte Rekapitulation der aktuellen Impfempfehlungen im Rahmen des Seminars, die folglich auch in das praktische Seminar integriert werden sollte.

## 1. Introduction

As future physicians, medical students will be responsible for imparting pertinent information and administering vaccines to patients. Physicians have a significantly higher credibility with patients when it comes to positive health behaviors if it is known whether or not they themselves are vaccinated [1]. There is greater probability that physicians who have been vaccinated against influenza will recommend the influenza vaccine to their patients than physicians who have not been vaccinated [2], [3]. The medical recommendations for a certain vaccine is in many cases significantly associated with the actual administration of that vaccine or at least the intention to receive it [4], [5], [6], [7], [8]: A higher percentage of the patients of physicians who are vaccinated against influenza are vaccinated against influenza than are the patients of unvaccinated physicians [8]. Despite the generally positive attitude of medical students toward vaccinations [9], their own protective immunity has gaps [10], [11], [12], [13], [14], [15], [16], [17], [18], [19], [20], [21] and does not have the necessary percentages to achieve herd immunity [22]. The National Catalogue of Competency-based Learning Objectives for Undergraduate Medical Education (NKLM), which was adopted in 2015, provides for interdisciplinary teaching of vaccination topics [http://www.nklm.de]: At graduation, medical students should, among other things, be able to weigh the risks and benefits of vaccinations, know the indications and contraindications, advise patients regarding immunization and administer vaccinations. The professional approach to immunization is based on three pillars: theoretical knowledge, practical skills and communication skills to inform about the importance of vaccinations and acknowledge the doubts and uncertainties of parents and patients. Medical students‘ knowledge of vaccinations is, however, incomplete [23], [24], [25]. Depending on the survey, only 39.8% to 77.9% of students are familiar with the general recommendations for healthcare workers regarding influenza vaccination [10], [25], [26]. Practical and communication skills in respect to the topic of vaccination are covered only late or not at all in a medical degree program.

At the medical school of the Goethe-Universität in Frankfurt am Main, the different aspects of immunization are integrated into many different courses. For instance, the scientific principles underlying immune system function are taught in the preclinical subjects of biology, biochemistry and physiology. During the clinical phase of study, additional theoretical bases for vaccination are included in lectures on microbiology and virology, general practice, internal medicine, and pediatrics. Focus is placed here on vaccine-preventable diseases, the types of vaccines and the corresponding vaccination schedules. However, when practicing medicine, a physician will be confronted with more or less well-documented vaccination certificates. The interpretation of such vaccination histories and the resulting determination of immune status and which vaccines are needed is not part of the medical curriculum.

Afonso et al. [27] have already been able to show that a two-hour, interactive vaccination seminar for first-semester medical students leads to a significant improvement in attitude toward the topic of immunization: Agreement with the statement, *“It is important to be vaccinated against Influenza”*, increased from 71% to 93% (p<0.01). Also, it was seen that students who were vaccinated against influenza viewed the influenza vaccination as more important than their unvaccinated peers did. In contrast to this US study, our study focuses on medical students at more advanced semester levels. Instead of administering vaccines, the practical focus was placed on making sense of vaccination certificates. The aim of our study was to investigate the extent to which a newly implemented vaccination seminar at the Goethe-Universität can contribute to improving students’ attitudes, knowledge, and practical skills in terms of understanding vaccination certificates. Furthermore, we wished to clarify if a theory-based seminar or a predominantly practice-based seminar is better suited to achieve this.

## 2. Project description

The vaccination seminar was integrated into the mandatory medical curriculum in cooperation with the Center for Internal Medicine (Zentrum der Inneren Medizin). Students complete a three-week-long block practicum in Internal Medicine during the second or third semester of clinical study. The first week of this practicum is the “central instruction week” and held as a seminar with practical exercises (e.g. ECG, sonography, doctor/patient consultations, evaluations of findings). Each semester, approximately 150 students attend this central week of instruction which takes place in small groups. For each small group, a one-hour time slot was found in which to hold the vaccination seminar. Due to the time constraint, it was necessary to limit the learning objectives and seminar content. Since administering vaccines is covered later in the block practicum on General Practice, we did not cover this topic in our seminar.

The following learning objectives were chosen:

- Students know the current vaccination recommendations of the STIKO (Standing Committee on Vaccination at the Robert Koch Institute in Berlin) and the different vaccination categories (standard, booster, indicated) and can recite this information;
- Students can identify indicated vaccines based on a vaccination certificate and correctly document an administered vaccine.

**Figure 1:**
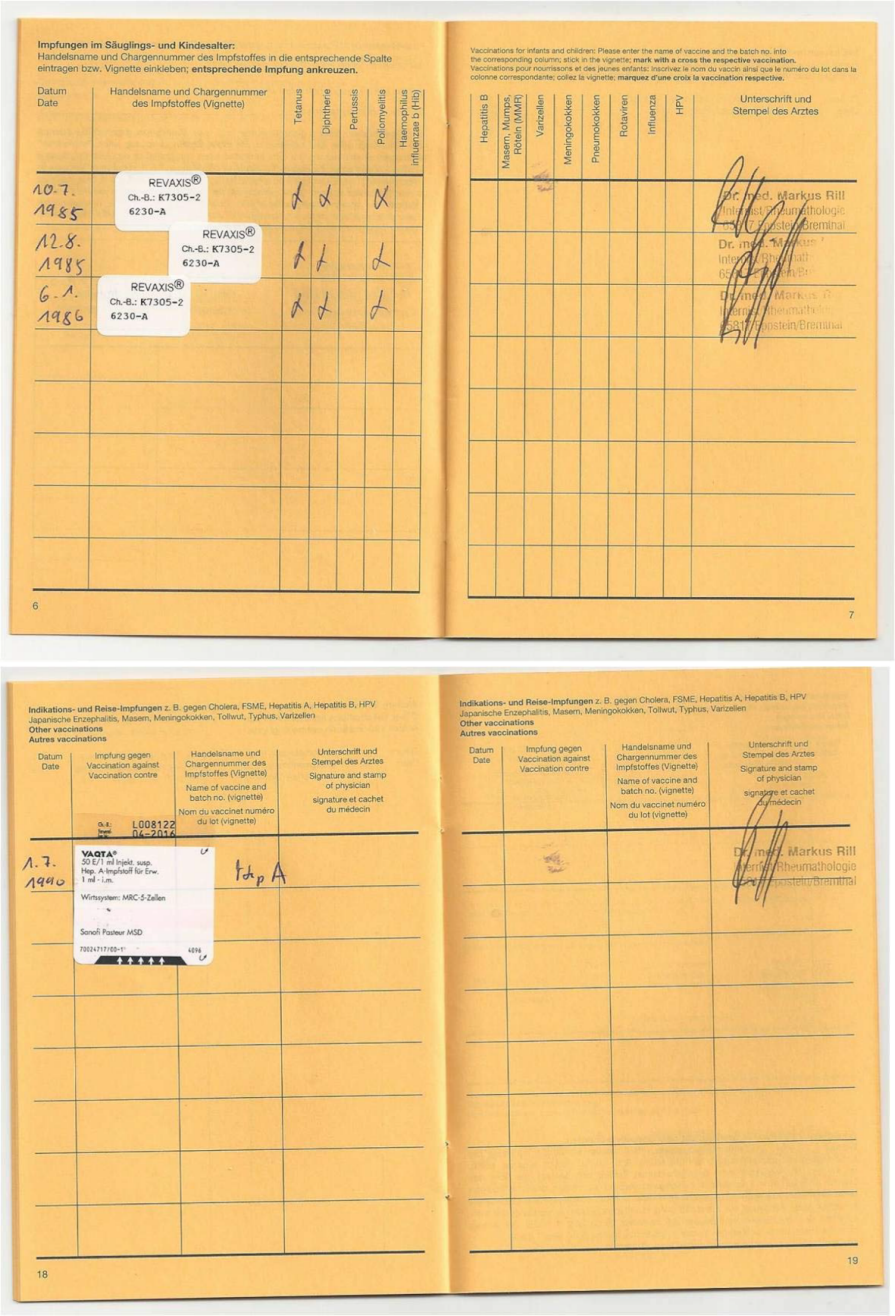
Course material for the practice-based seminar – fictive vaccination certificate for “Polly Ester” Anamnesis: Polly Ester is 30 years old. She would like to get pregnant and wants to have her health status checked so as not to cause damage to the child. As a child she had a strong reaction to a vaccination, the reason why her mother did not allow her to be immunized further. Polly Ester can no longer recall which vaccination caused the reaction or how old she was when it happened. Task: Does the patient have a complete standard immunity? If not: Which catch-up vaccinations need to be given to achieve the standard protective immunity? Are additional vaccinations necessary or a good idea?

During the 2017/18 winter semester, 34.2% of the students were taught in a theory-based seminar; the remaining 65.8% in a practice-based seminar. The theoretical seminar consisted of a lecture on the principles and theoretical aspects of immunization, contraindications, side effects, and possible complications. The STIKO recommendations, communicating with patients about vaccinations, and correct documentation of a vaccination were also covered in this seminar. In the practice-based seminar, the learning content was imparted using four fictive patient cases reflecting different vaccination-related concerns. The students were given a fictive vaccination certificate and the corresponding patient’s medical history. Groups of three to four students were assigned the task of evaluating the status of the patient’s immunity based on the STIKO recommendations. Figure 1 (Fig. 1) contains one of the example patient cases used in this seminar.

**Figure 2:**
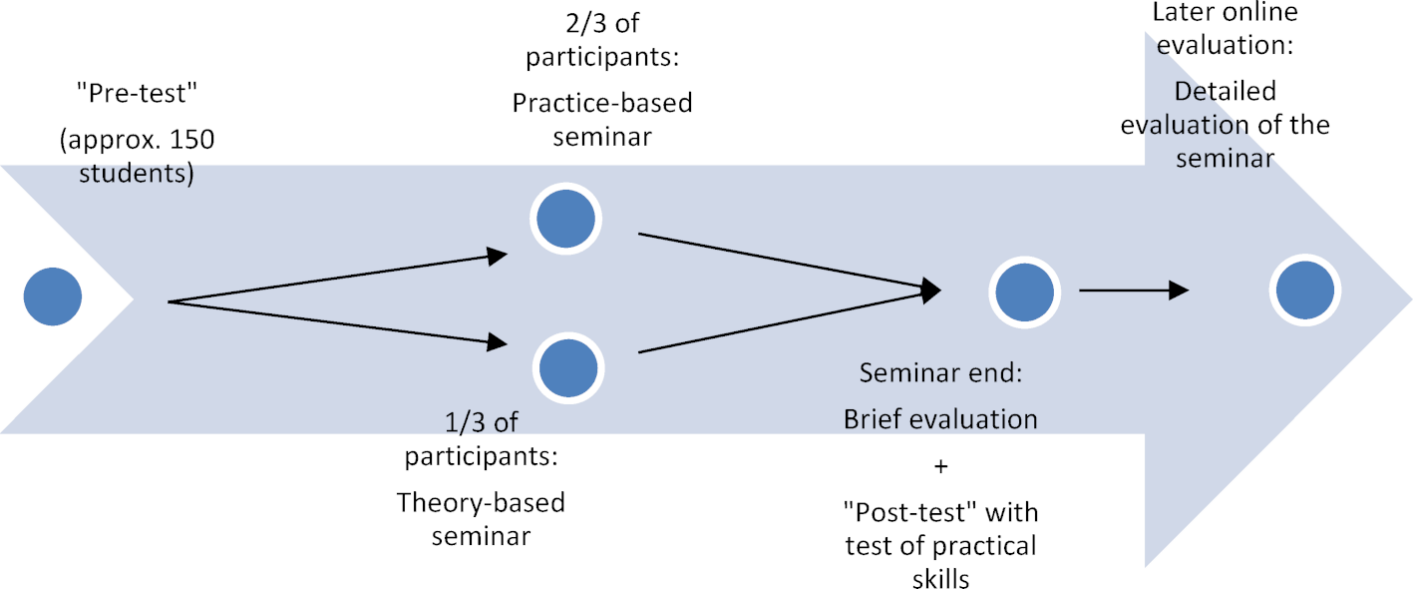
Timeline for the different surveys

**Figure 3:**
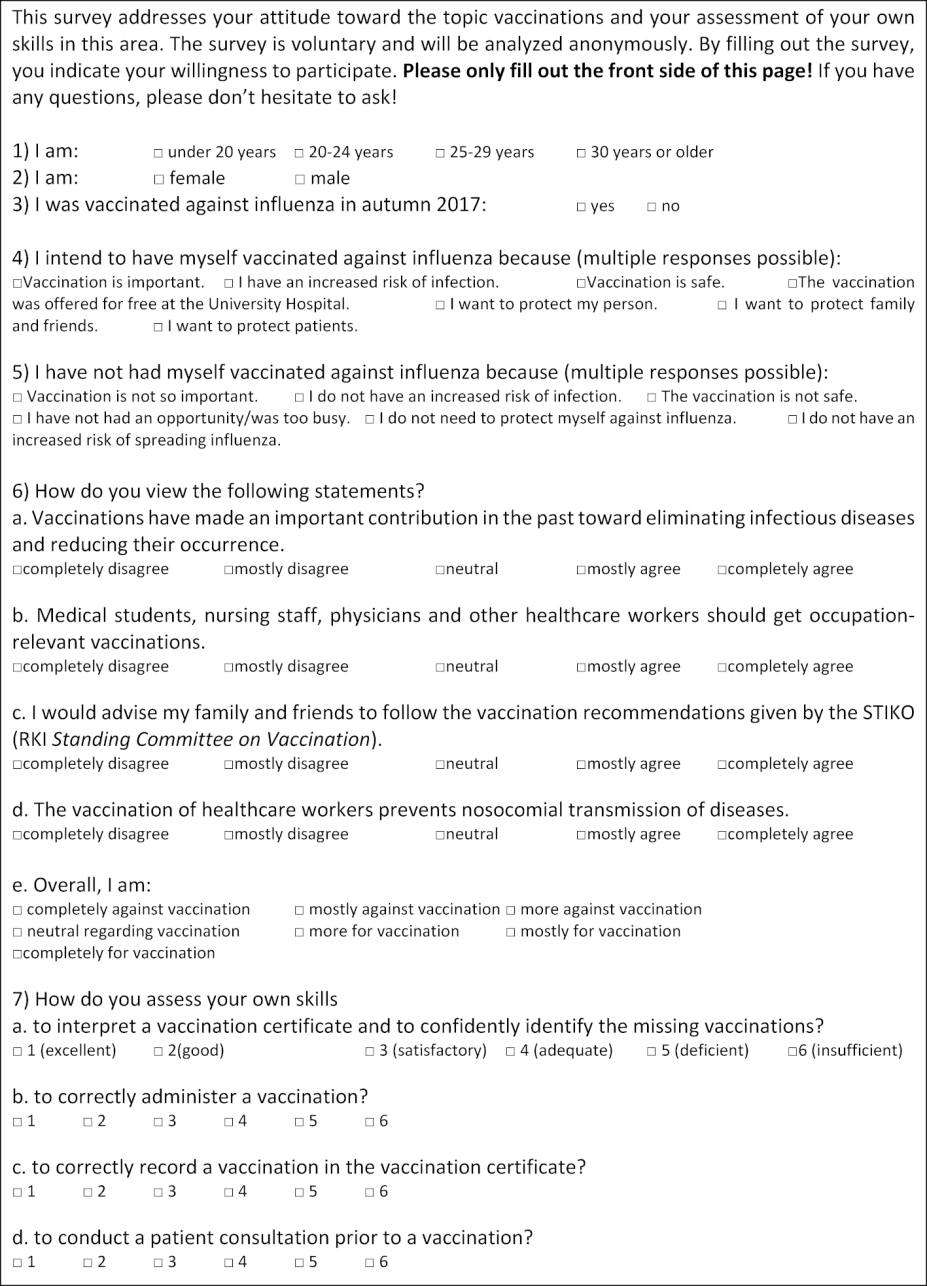
Survey 2017/18 Winter Semester (front side)

**Figure 4:**
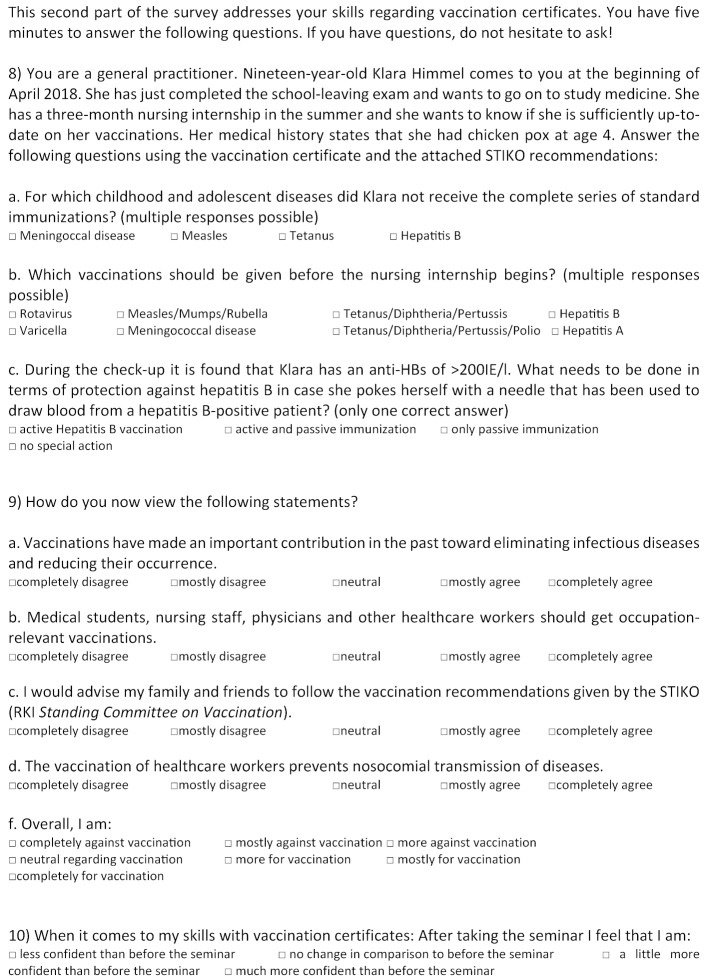
Survey 2017/18 Winter Semester (reverse)

Student surveys were conducted at different time points – before and directly after the seminar and after conclusion of the first week of the practicum; an overview is presented in figure 2 (Fig. 2). At the beginning of the seminar a voluntary and anonymous student survey was conducted using a questionnaire (“pre-test”). This pre-test (see figure 3 (Fig. 3)) asked for demographic information (age, gender), influenza vaccination status in the 2017/18 flu season, and questions about the three focal areas of this study (attitude, knowledge, practical skills). A total of four questions were asked about attitude toward vaccinations (see figure 3 (Fig. 3), questions 6a-6c, 6e); the responses were given on five-point or seven-point Likert scales. Questions were also asked about the reasons for and against an influenza vaccination (see figure 3 (Fig. 3), questions 4, 5). The pre-test contained a question on knowledge (see figure 3 (Fig. 3), questions 6d): Students were asked to rate their level of agreement with the statement, “The vaccination of healthcare workers prevents nosocomial transmission of diseases,” on a five-point Likert scale. To gather data on practical skills prior to the seminar, students were asked to assess their own practical skills regarding immunization (e.g. ability to identify necessary vaccinations on the basis of a vaccination certificate) using the conventional German academic grading scale (see figure 3 (Fig. 3), questions 7a-d). The “post-test” was administered after completion of the seminar (see figure 4 (Fig. 4)); the same questions were asked again about attitude and knowledge. To measure practical skills, a fictive vaccination certificate was also handed out as part of the post-test. Students were asked to determine which series of standard immunizations had not been fully completed and to identify additional indicated vaccines for the fictional patient (a secondary school graduate prior to beginning a nursing internship). The answers were recorded using single- or multiple-choice responses. As a final question, students were asked if they now felt more confident in understanding and interpreting vaccination certificates. A brief evaluation was conducted at the conclusion of the seminar giving students the opportunity to rate the seminar based on the German grading scale. A link to the voluntary online evaluation was then sent to the participants after conclusion of the first practicum week. Within the scope of this final survey, there was a more detailed evaluation of the seminar (e.g. relevance to future medical practice);table 1 (Tab. 1) contains the questions of the online evaluation.

Statistical analysis was performed using the software program “BiAS.” for Windows (program version 11.02). The test methods applied include: chi^2^ test, Mann-Whitney U test, Wilcoxon matched pairs test, Spearman rank correlation, Friedman test.

An “agreement score” was calculated to better analyze the questions about attitude by adding the individual point values for the responses on the Likert scale to yield a score that reflects the agreement with the vaccination topic; values ranged from a minimum of 4 to a maximum of 22 points. This score is not to be confused with the “overall attitude” of the students which represents a subjective assessment by the students (response to the statement: *“Overall, I am completely for/mostly for/more for/neutral/more against/mostly against/completely against vaccination”*). To better differentiate between attitudes, the response options were placed on a seven-point Likert scale, rather than a five-point one.

To analyze students’ skill in handling vaccination certificates, a “vaccination certificate score” was calculated, with a possible maximum of 12 points. To calculate this, a point was given for each correctly ticked box on the two post-test questions about a specific vaccination certificate; a point was also given for each box that was correctly left unticked. The scores were developed in collaboration with a colleague at the Institute for Biostatistics and Mathematical Modeling at the Goethe-Universität Medical School.

## 3. Results

Of the 149 seminar attendees, 148 participated in the study (99.3%). Table 2 (Tab. 2) provides an overview of the participants’ demographic data and their vaccination status. The distribution according to gender, age groups, and those vaccinated against influenza did not significantly differ from each other in the two seminar formats.

### 3.1. Attitude

**Figure 5:**
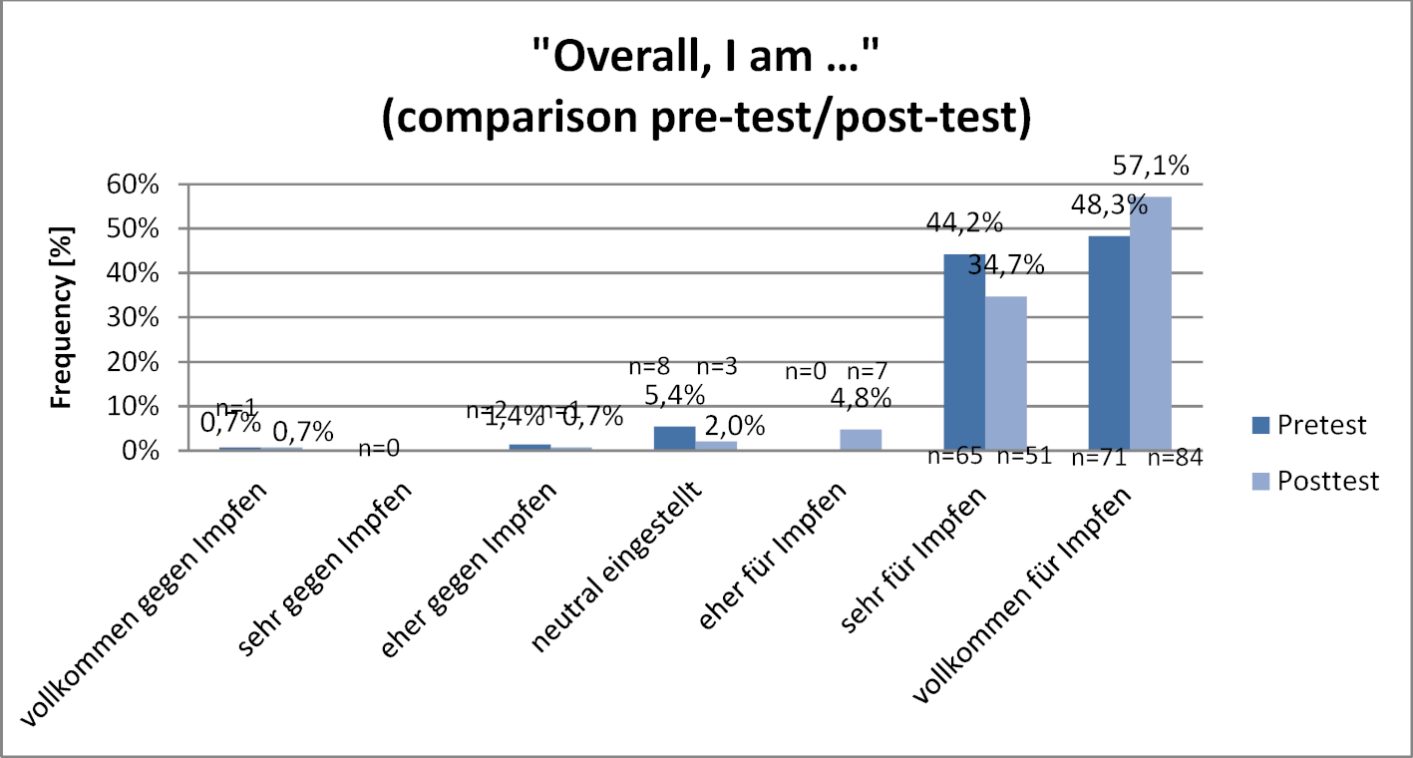
General attitudes of the students toward the topic of vaccinations (comparison pre-test/post-test) Subjective self-assessment of the students before and after the seminar (response to the question: “Overall I am completely for/mostly for/more for/neutral/more against/mostly against/completely against vaccination”). n=147

**Figure 6:**
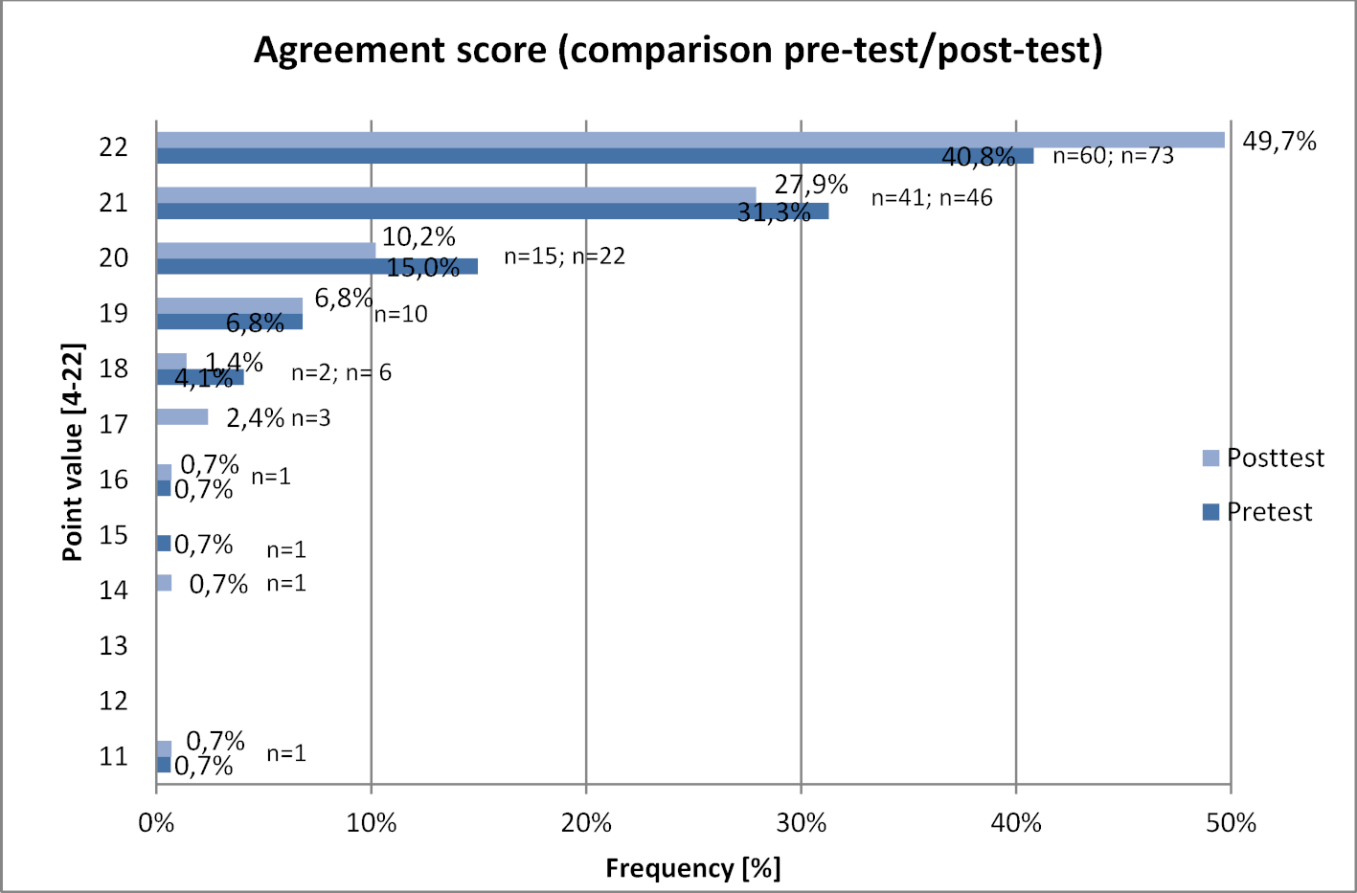
Attitudes of the students — Agreement score (comparison pre-test/post-test) To better analyze attitudes toward the topic of vaccination, an “agreement score” was calculated from the responses to the four questions about attitudes by adding the individual point values of the responses on the Likert scale to yield a score that reflects the agreement with the vaccination topic, with values ranging from a minimum of 4 to a maximum of 22 points.

Students generally have a positive attitude toward vaccinations. Primarily among the students who had a very positive attitude prior to the seminar, it was possible to improve this further as a result of the seminar. Students who are vaccinated against influenza have a higher level of agreement than those who are not vaccinated. At the beginning of the seminar, 92.5% (n=136) of the students indicated that they were overall “more for,” “mostly for,” or “completely for” vaccination; in the post-test it was 96.6% (n=142) (see figure 5 (Fig. 5)). For the agreement score (see figure 6 (Fig. 6)), the pre-test had a median of 21 points (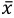 20.86; SD 1.51), likewise in the post-test (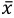 20.99; SD 1.59). A tendential increase is visible with p=0.07, even if it is without statistical significance. If only the students who already showed a high level of agreement in the pre-test (agreement score ≥18 points) were included in the analysis, there is a significant increase in the agreement score from 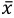 21.06 in the pre-test (SD 1.05; median 21) to 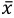 21.23 in the post-test (SD 1.00; median 22) with p=0.01. The agreement of the students with a lower agreement score before the seminar did not change after the seminar. Table 3 (Tab. 3) presents the analysis of the agreement score according to seminar format, gender, age, and vaccination status: No significant differences could be detected in reference to the attitudes of the participants in the different seminar formats, age groups or between the two genders. However, there are differences between the students who are vaccinated against influenza and those who are not. Students vaccinated against influenza have a significantly higher agreement score than unvaccinated students. The agreement score and overall attitude of the vaccinated students did not improve further as a result of the seminar. Nonetheless, the group of unvaccinated students did tend (p=0.051) to have a higher agreement score, and while the overall attitude of this group significantly (p=0.04) improved as a result of the seminar (see table 4 (Tab. 4)), it still remained behind the overall attitude of the vaccinated students.

#### 3.2. Knowledge

Students‘ knowledge of vaccination topics could be increased by the seminar, regardless of format, age, gender or influenza vaccination status: In the pre-test, 115 students (79.3%) completely agreed or mostly agreed with the statement, *“The vaccination of healthcare workers prevents nosocomial transmission of diseases”*; in the post-test the number was 128 students (86.4%) (see table 5 (Tab. 5), section a). In the pre-test there was a mean value of 4.0 on the five-point Likert scale; in the post-test the mean value was at 4.28. This difference is statistically significant with p<0.001. For the group of vaccinated students, agreement with this statement increased more strongly than for the unvaccinated group (see table 5 (Tab. 5), section b): The mean value for agreement on the five-point Likert scale increased from 3.97 to 4.4 (p<0.001; R=0.67). For the unvaccinated students, the mean value increased from 4.04 to 4.19 (p=0.06; R=0.29).

#### 3.3. Practical skills

The students assessed their skills in handling vaccination certificates as being “good” to “satisfactory.” The assessment of whether they could evaluate a vaccination certificate or identify the missing vaccinations was significantly (p<0.001) poorer than their assessment of other skills (administering and documenting a vaccination). The self-assessments of the students aged at least 25 years old and the students vaccinated against influenza were in part significantly better than the self-assessments of the younger or unvaccinated students (see table 6 (Tab. 6)). In the post-test the students achieved an average vaccination certificate score of 8.76 points (SD 1.36). Table 7 (Tab. 7) shows the vaccination certificate score for the different groups. Participants in the theory-based seminar tended to score better with an average of 8.92 points (SD 1.34) than participants in the practice-based seminar (8.68 points; SD 1.38); however, this difference was not statistically significant (p=0.36). Age and influenza vaccination status also showed no significant influence on the vaccination certificate score. Participants who attended the practice-based seminar made mistakes significantly more often (p=0.04) than participants in the theory-based seminar and misidentified primary vaccinations as missing when they were not.

#### 3.4. Self-assessment

**Figure 7:**
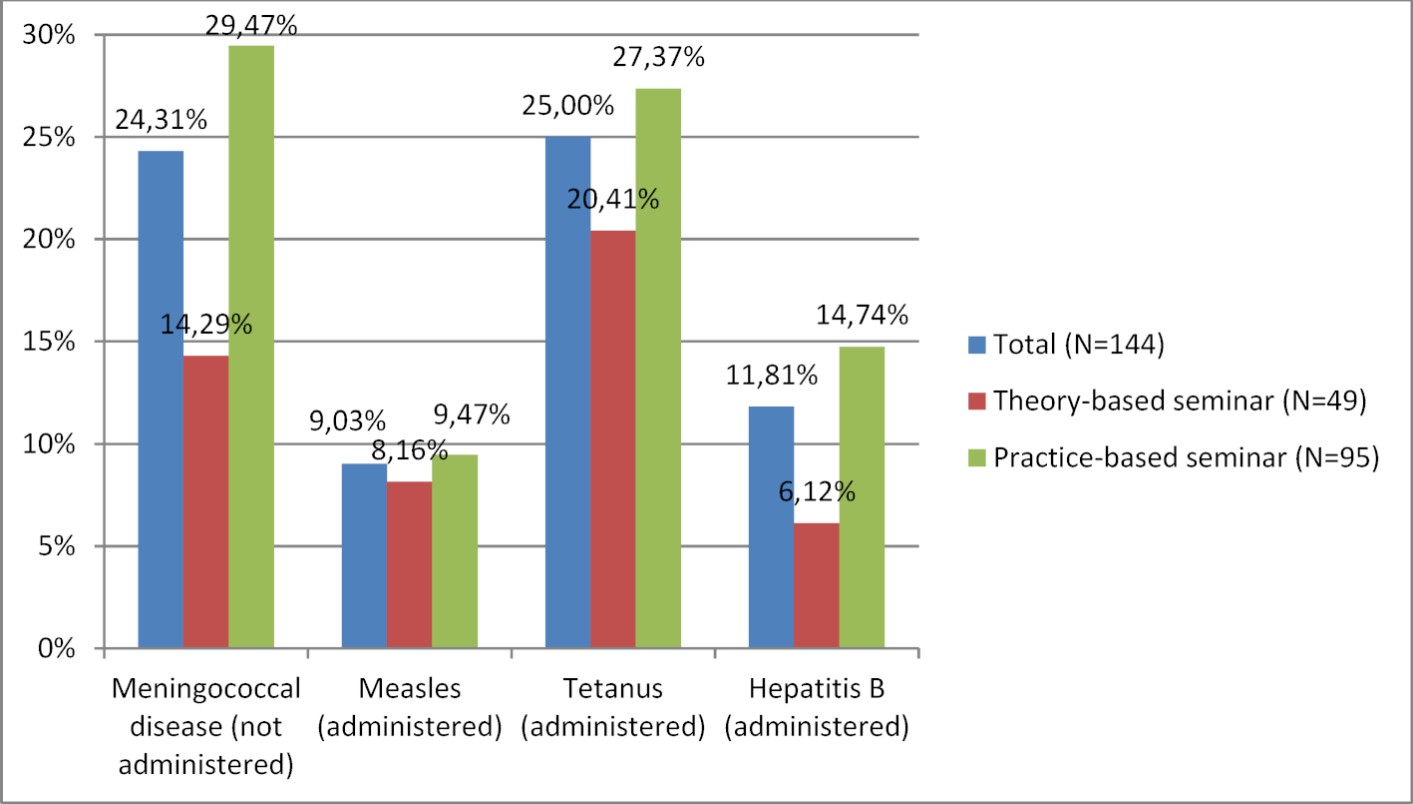
Using vaccination certificates — Wrong answers to the question: “For which childhood and adolescent diseases did Klara not receive the complete series of standard immunizations?” Given in percentages. Participants in the theory-based seminar misidentified vaccines as missing on an average of 0.41 (SD 0.67), even though they had been given. Participants in the practice-based seminar misidentified vaccines as missing on an average of 0.72 (SD 0.87), even though they had been given. This difference is statistically significant with p=0.04.

No student felt less confident after the seminar than before when it came to understanding and working with vaccination certificates. A total of 72.6% felt a little more confident and 17.8% much more confident than before the seminar. Participants who attended the practical seminar felt more confident after the seminar than participants who attended the theoretical seminar (p<0.01). There was no correlation between a perception of greater confidence in handling vaccination certificates and the vaccination certificate score. Furthermore, contradictory results were visible regarding the self-assessment and the actual skills demonstrated: The participants who attended the practice-based seminar misidentified primary vaccinations as missing when they were not, despite having a greater feeling of confidence. Still, students with a high level of confidence recommended significantly fewer vaccinations that were not in fact indicated (see figure 7 (Fig. 7)).

#### 3.5. Evaluation

The online evaluation was completed by 27 students (18% of participants). Sixteen (59.3%) attended the practice-based seminar and 11 (40.7%) the theory-based seminar, a distribution which corresponds with the assignment of the students to the two teaching formats. The participants in the theoretical seminar gave an average rating of 2.9 (SD 1.0; median 3); the practical seminar was rated on average with 1.9 (SD 0.7; median 2). This difference is statistically significant (p=0.02; R=0.5; Mann-Whitney U test).

## 4. Discussion

To our knowledge, this study is the first of its kind in the German-speaking countries to investigate the influence of a vaccination seminar on the attitude, knowledge and practical skills of medical students. In 2011/12, Afonso et al. [27] held a two-hour vaccination seminar for first-semester medical students and were able to significantly improve the students’ attitude toward the topic of immunization as a result. It was also shown that students who were vaccinated against influenza viewed the influenza vaccine as more important than their unvaccinated peers did. In contrast to this US study, our study focused on medical students in the later clinical phase of study. The practical focus was placed on understanding and using vaccination certificates. Our study was also able to determine an improvement in the attitude of medical students toward the topic of vaccination. Since at the beginning of the seminar 92.5% of the students were already overall “mostly for” or “completely for” vaccination, the increase was not so dramatic than that seen in Afonso et al. A significant improvement could be shown only for the group of students who already had a high level of agreement with pre-formulated statements at the beginning of the seminar. Our study also shows that students who are vaccinated against influenza have a significantly higher level of agreement with pro-vaccination statements than unvaccinated students. The attitude of unvaccinated students improved significantly as result of the seminar, but remained behind the attitude of the students who were vaccinated against influenza. Influenza vaccination status can be seen as an expression of a positive attitude toward vaccinations [28]: Vaccinated individuals view protective immunity against influenza as important enough to make the effort to get vaccinated. Conversely, having a positive attitude toward vaccination does not automatically result in getting vaccinated against influenza: Over 90% of the students have a very positive attitude toward immunization, but only less than half are vaccinated against influenza. In 1975 Ajzen & Fishbein first described their theory of reasoned action postulating that human behavior is determined by intention to act which, in turn, is influenced by personal attitudes and social norms [29]. The discrepancy between the intended and actual behaviors is referred to as the intention-behavior gap [30]. There are probably different reasons for the discrepancy uncovered in our study between having a positive attitude toward vaccination and actual influenza vaccination behavior on the part of the students. A survey conducted by Petersen et al. showed, for instance, that only 46.4% of the surveyed students knew of the general recommendation that healthcare workers be vaccinated against influenza [10]. Many students judge the importance, safety [31] and efficacy [32], [33] of the influenza vaccination as deficient.

The data in this study allow speculation that students’ gain in knowledge is higher if at the beginning of the seminar they already have a positive attitude: The agreement with the factually correct statement, *“The vaccination of healthcare workers prevents nosocomial transmission of diseases,”* could be increased by the seminar. A significant increase could be observed among the students who were vaccinated against influenza; in the group of unvaccinated students the increase in agreement with this statement was not significant. The practice-based seminar was rated significantly better than the theory-based seminar and the content was viewed significantly more often as being relevant to future medical practice. Participants who attended the theory-based seminar tended to score higher on understanding vaccination certificates than those who attended the practice-based seminar. This is possibly due to the fact that it is easier for students to recall knowledge if it has been previously reviewed by means of structured teaching. A review of the current vaccination recommendations took place during the theoretical seminar; in the practical seminar the recommendations were handed out but not discussed.

The percentage of women in the theory-based seminar was 76.5% and 60.8% in the practical seminar. No statistically significant difference in connection with the gender distribution between the two course formats was found using the chi^2^ test (p=0.08). Since the agreement score in the pre-test did not differ between the genders, we are not assuming an influence of gender on the different results for the two seminar formats.

Although the participants attending the practice-based seminar felt significantly more confident after the seminar than the participants attending the theoretical seminar, they did not score higher when it came to interpreting and understanding the vaccination certificate. No correlation was found between a high degree of perceived confidence with vaccination certificates and the vaccination certificate score; however, students with a greater feeling of confidence recommended non-indicated vaccinations significantly less often. These results are indeed contradictory. One explanation for this could be a lack in the students’ ability to self-assess, although a weakness in the questionnaires concerning the questions on practical skills cannot be ruled out.

### Limitations and strengths of the study

At the beginning of the seminar the students were informed and asked to fill out the survey. In the case that students were not interested in participating, they were asked to turn in the survey at the same time as the willing participants to ensure the voluntary and anonymous nature of the survey. Despite this, it cannot be ruled out that responses were given in an effort to produce the socially desired ones. To gain the most unbiased picture possible of the actual attitudes of students, multiple surveys were conducted and a score was calculated based on all of them. A biased depiction of the actual attitude is, however, still conceivable. For reasons pertaining to data protection, the seminar evaluation had to take place as an online survey and was not integrated into the official course evaluation for the first week of the block practicum. This was certainly disadvantageous for the response rate: Only 27 students (18% of participants) completed the online evaluation. Among the strengths of this study is the required participation of all medical students in a vaccination seminar. As a result, it was possible to capture a comprehensive view of student attitudes and knowledge. An elective vaccination seminar would probably have been attended primarily by students interested in the topic of immunization and would have yielded even more positive attitudes than are actually present in the full student cohort.

## 5. Conclusion

The observations of this study lead to the assumption that among medical students at the time of the seminar there were already fixed views on vaccinations that can only be changed minimally by a seminar with limited time for instruction. Questions on personal views are often loaded with emotional components. Precisely with the topic of immunization is this strongly pronounced, something which is repeatedly evident in public debates between those for and those against vaccination [34]. It must be assumed that, even among medical students who view immunization skeptically, the opinions and stances on topics related to vaccination are grounded more in emotion than in science. There is a discrepancy between the positive attitudes and the very prevalent intention to get vaccinated against influenza and the actual vaccination behavior. Despite the easy availability of information and opportunities to receive the annual influenza vaccine at the University Hospital in Frankfurt, less than half of the students are vaccinated. It remains to be seen if the implementation of the vaccination seminar contributes in the long term to an increase in the vaccination rate for influenza or other occupationally indicated vaccines. Further research should look at which measures are effective in turning the positive attitude toward vaccinations and the real intentions to be vaccinated against influenza into high rates of actual vaccination against influenza. To compensate for the deficiency seen in the participants who attended the practice-based seminar regarding their ability to interpret and understand vaccination certificates, a structured review of the STIKO recommendations should be integrated into the practice-based seminar prior to the group work with fictive vaccination certificates.

## Acknowledgements

We wish to thank Prof. Ochsendorf (MME, Dermatology Clinic, University Hospital, Frankfurt) for his valuable advice on the design for the seminar and the study. Our thanks also go to Mr. Scherzer (Teaching Office, Internal Medicine) for providing the evaluation data and assisting with the organization of the seminar. Finally, we wish to express our gratitude to all of the students who participated in the seminar and this study.

## Competing interests

Sabine Wicker is a member of the Standing Committee on Vaccination. The authors declare that there are no financial or economic conflicts of interest.

## Figures and Tables

**Table 1:**
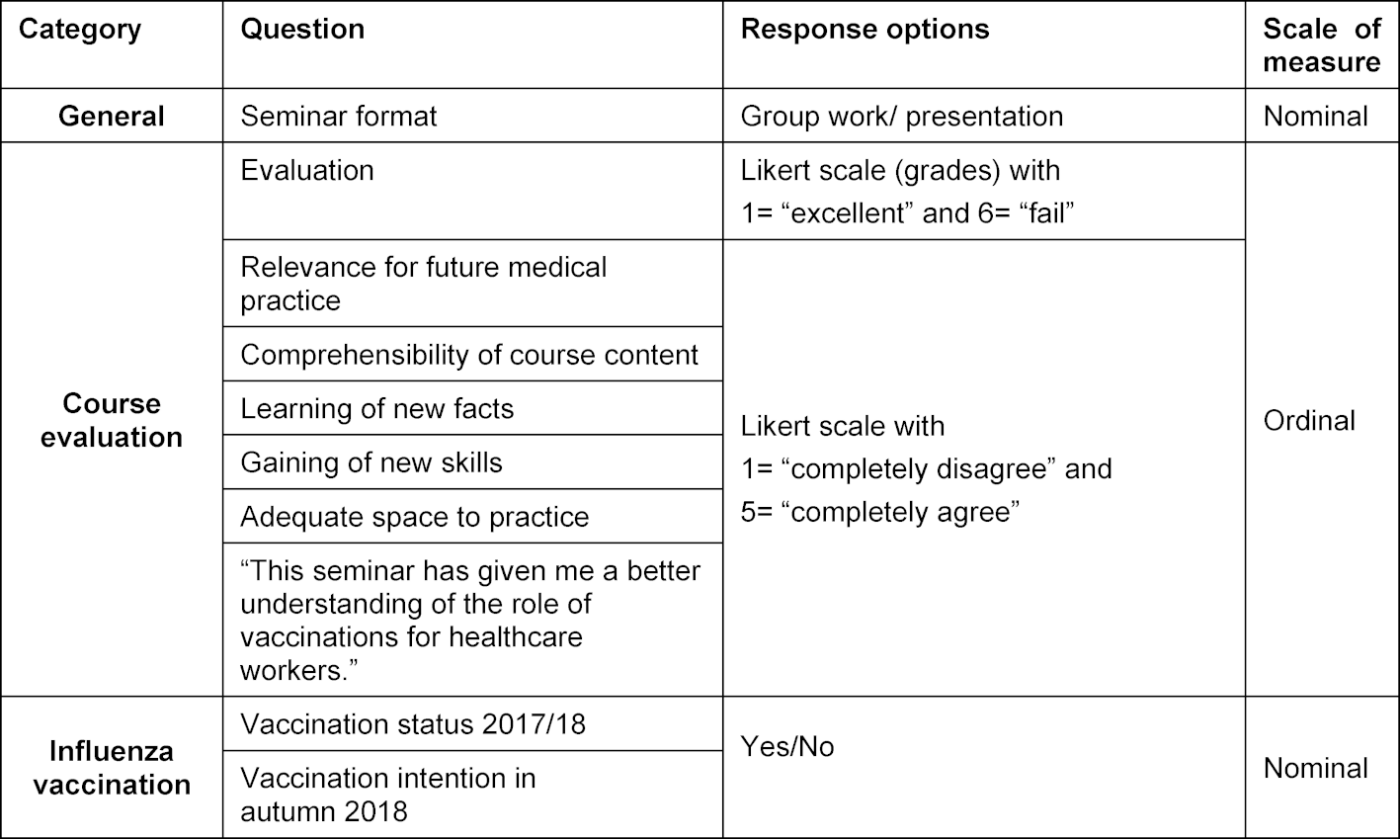
Online evaluation, winter semester 2017/18. Overview of questions, response options and scale of measure

**Table 2:**
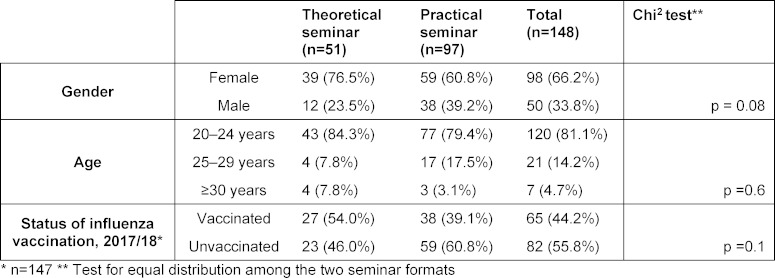
Demographic data on the participants in the winter semester 2017/18

**Table 3:**
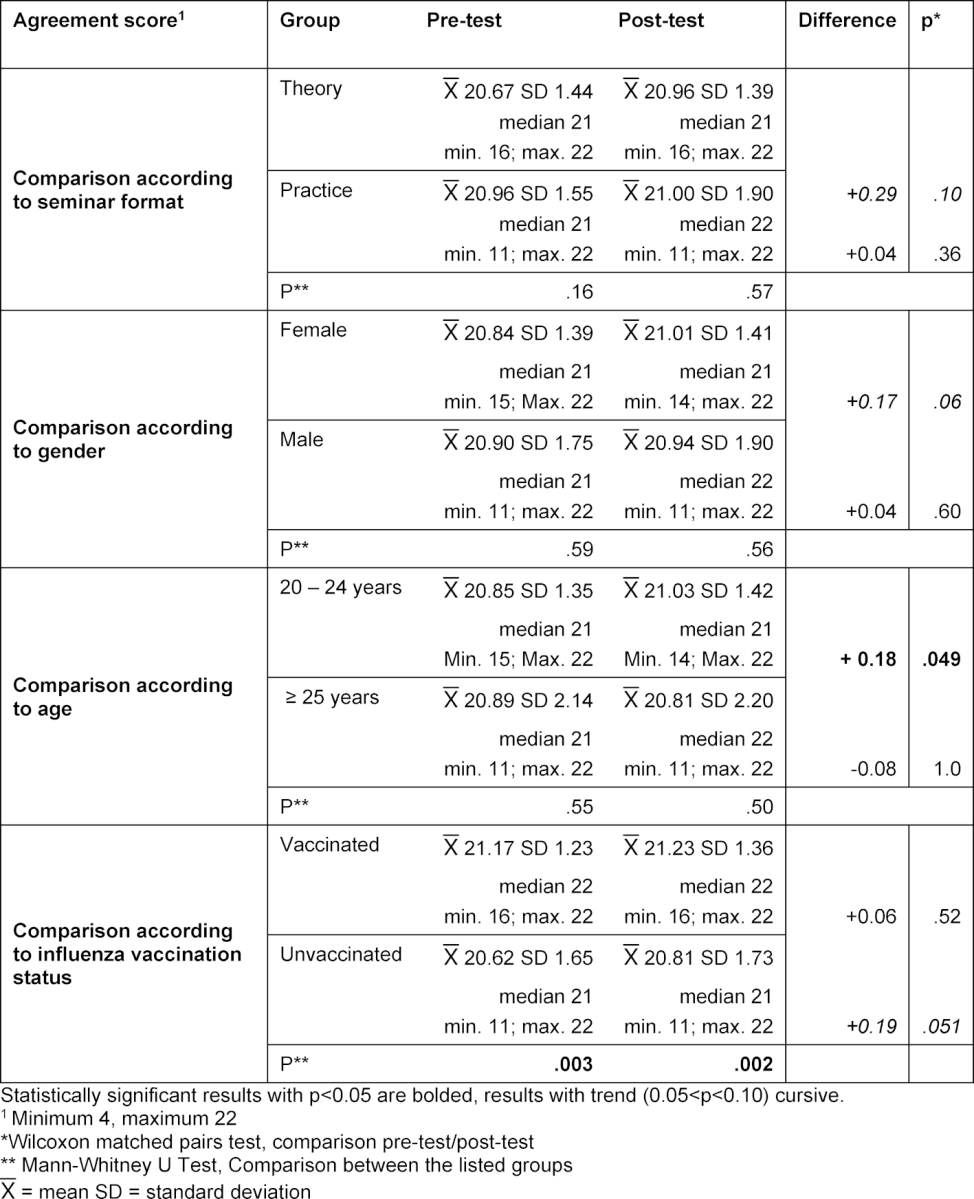
Attitudes toward the topic of vaccinations – Agreement score. To better analyze attitudes toward the topic of vaccination, an “agreement score” was calculated from the responses to the four questions about attitudes: Adding the individual point values of the responses on the Likert scale yields a score that reflects the agreement with the vaccination topic, with values ranging from a minimum of 4 to a maximum of 22 points. Here, the agreement score is compared according to seminar format, gender, age, and vaccination status.

**Table 4:**
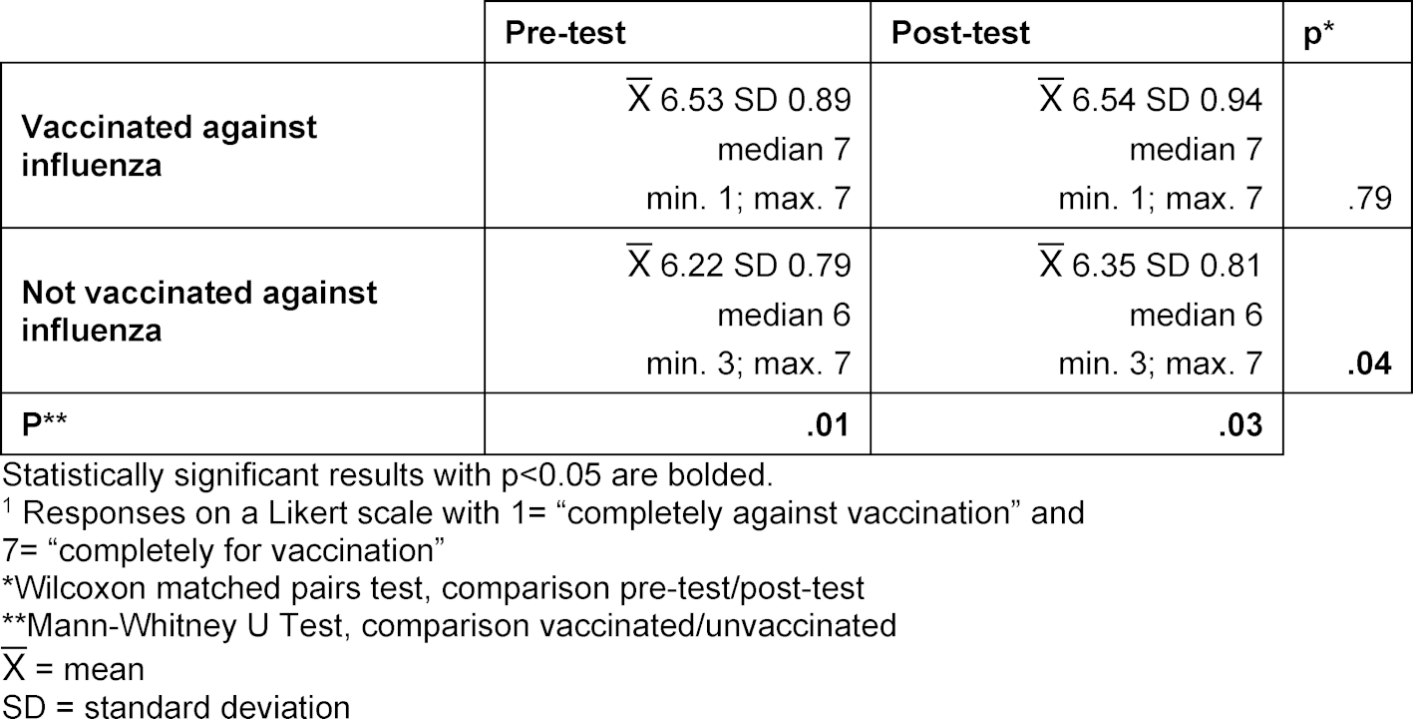
Attitude toward the topic of immunization – Comparison of the overall attitude according to vaccination status. Subjective assessment of the students of their own overall attitude (response to the question, *“Overall, I am completely for/mostly for/more for/neutral/more against/mostly against/completely against vaccination”^1^*) before and after the seminar (pre-test/post-test) depending upon vaccination status.

**Table 5:**
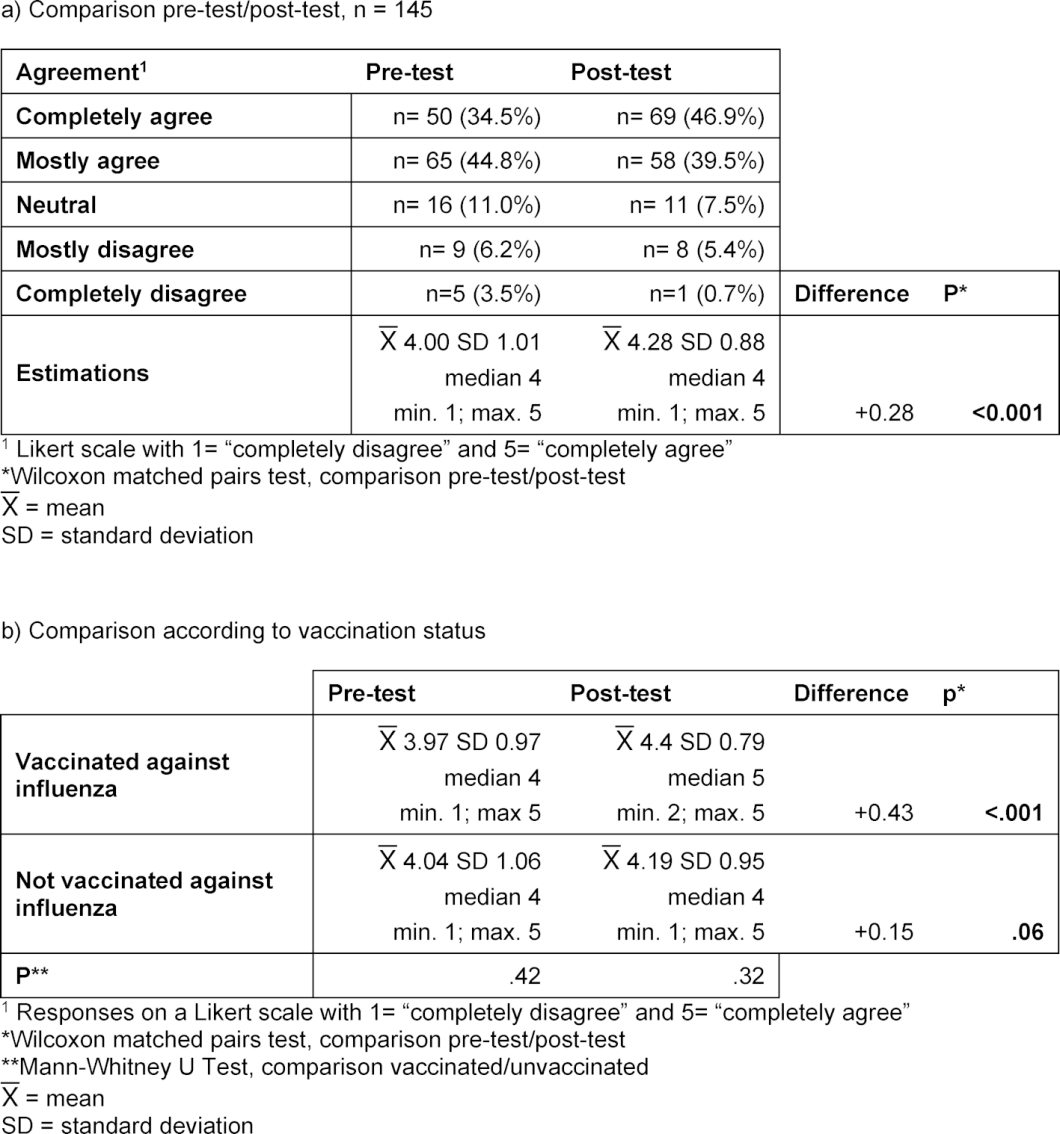
Medical students’ knowledge — Agreement with the statement, *“The vaccination of healthcare workers prevents nosocomial transmission of diseases.”*

**Table 6:**
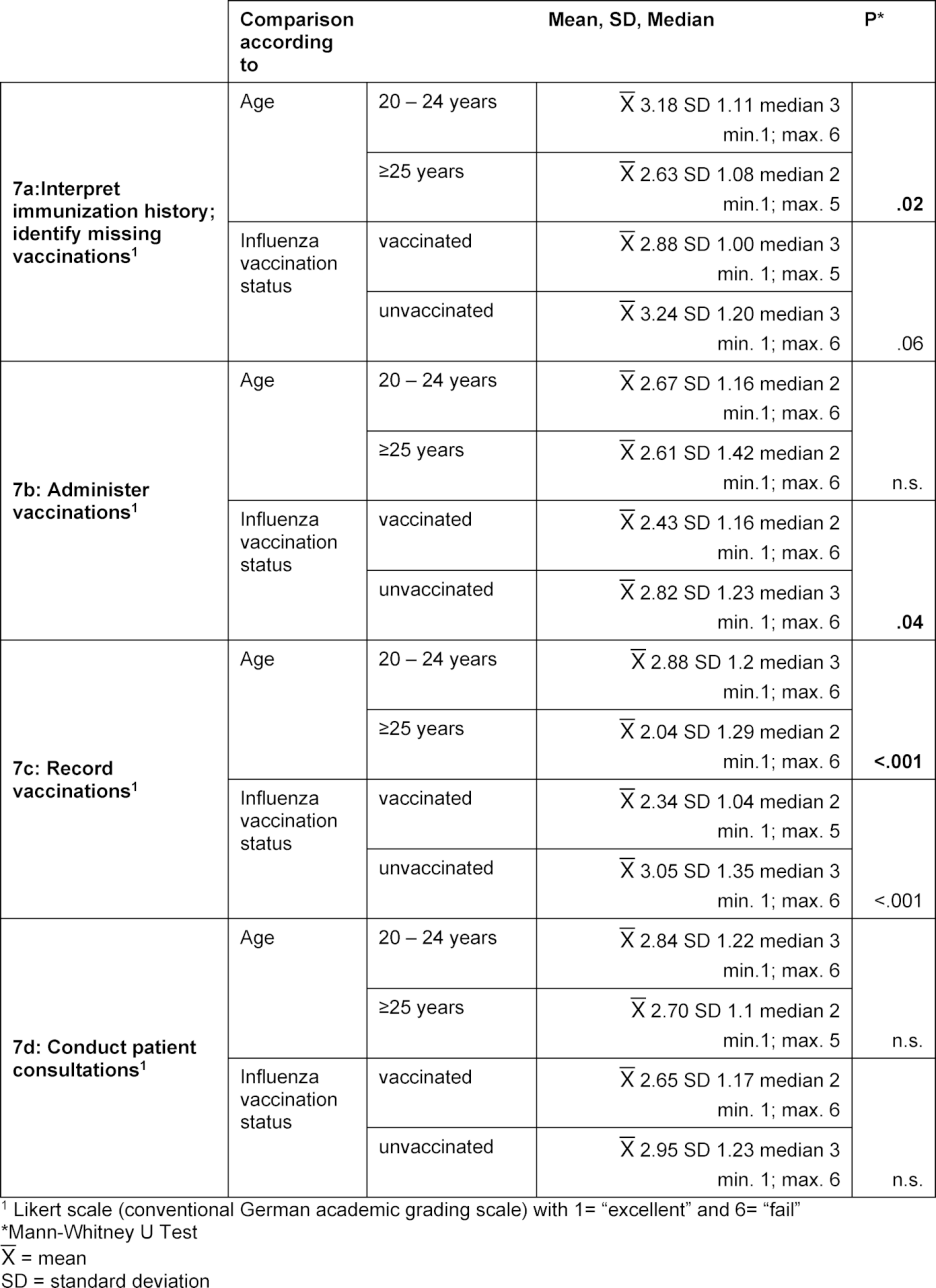
Practical skills — Self-assessment of the students. Comparison according to age group and influenza vaccination status

**Table 7:**
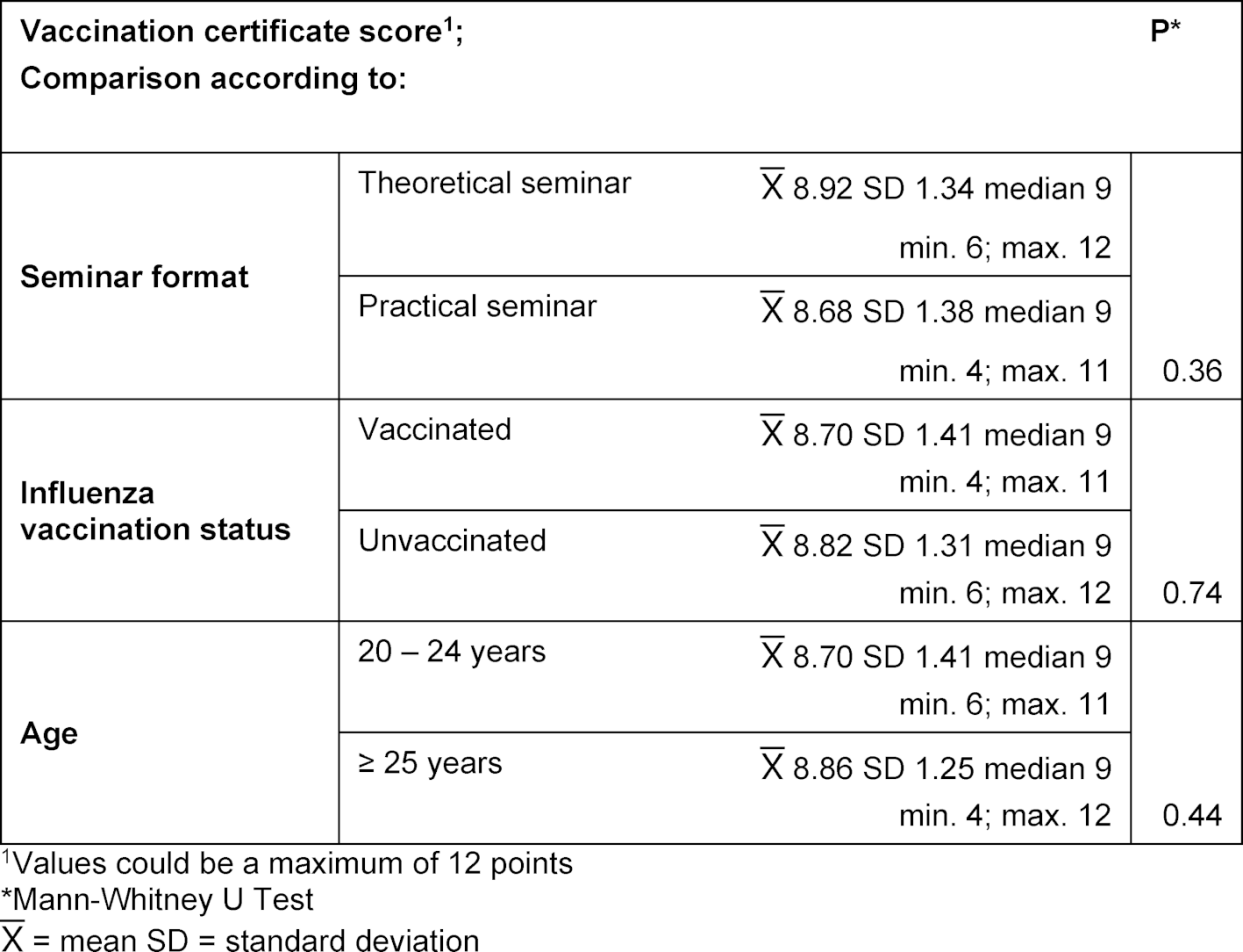
Practical skills — Understanding vaccination certificates and vaccination certificate score. Comparison according to seminar format, vaccination status, age group
